# Oncolytic viruses-modulated immunogenic cell death, apoptosis and autophagy linking to virotherapy and cancer immune response

**DOI:** 10.3389/fcimb.2023.1142172

**Published:** 2023-03-15

**Authors:** Yi-Ying Wu, Te-Kai Sun, Ming-Shan Chen, Muhammad Munir, Hung-Jen Liu

**Affiliations:** ^1^ Institute of Molecular Biology, National Chung Hsing University, Taichung, Taiwan; ^2^ The iEGG and Animal Biotechnology Center, National Chung Hsing University, Taichung, Taiwan; ^3^ Tsairder Boitechnology Co. Ltd., Taichung, Taiwan; ^4^ Department of Anesthesiology, Ditmanson Medical Foundation Chia-Yi Christian Hospital, Chia-Yi, Taiwan; ^5^ Department of Biomedical and Life Sciences, Lancaster University, Lancashire, United Kingdom; ^6^ Rong Hsing Research Center for Translational Medicine, National Chung Hsing University, Taichung, Taiwan; ^7^ Ph.D Program in Translational Medicine, National Chung Hsing University, Taichung, Taiwan; ^8^ Department of Life Sciences, National Chung Hsing University, Taichung, Taiwan

**Keywords:** oncolytic viruses, virotherapy, immunotherapy, apoptosis, immune system, autophagy, immunogenic cell death

## Abstract

Recent reports have revealed that oncolytic viruses (OVs) play a significant role in cancer therapy. The infection of OVs such as oncolytic vaccinia virus (OVV), vesicular stomatitis virus (VSV), parvovirus, mammalian reovirus (MRV), human adenovirus, Newcastle disease virus (NDV), herpes simplex virus (HSV), avian reovirus (ARV), Orf virus (ORFV), inactivated Sendai virus (ISV), enterovirus, and coxsackievirus offer unique opportunities in immunotherapy through diverse and dynamic pathways. This mini-review focuses on the mechanisms of OVs-mediated virotherapy and their effects on immunogenic cell death (ICD), apoptosis, autophagy and regulation of the immune system.

## Introduction

Oncolytic viruses (OVs) are new class of natural means of immunotherapy and hold promising future prospects in oncology. OVs are being evaluated as immunotherapies for a variety of advanced malignancies. These have been proposed as potential cancer treatments by inflaming the tumor microenvironment (TME) and directly killing tumor cells. The oncolytic HSV in immunotherapy cracks tumor cells without causing damage to normal cells (Ma et al., 2018). The therapeutic promise of OVs depends on their ability to selectively kill tumor cells and induce antitumor immunity while protecting healthy cells. OVs-mediated tumor destruction is now recognized as a powerful tool to aid the development of anti-tumor immunity (Glorioso et al., 2021). The perfect OV should eliminate cancer cells through a combination of three mechanisms, including the induction of apoptosis, pro-inflammatory cytokines, and interferons (Schiller et al., 2012; Mahmoud et al., 2015; Frumence et al., 2016). In addition to directly killing tumor cells, OVs activate immune responses or express healing factors to elevate antitumor efficacy, thereby enhancing efficacy of cancer immunotherapies (Sobhanimonfared et al., 2020; Yang et al., 2021). Additionally, OVs-mediated apoptosis may trigger anticancer immune responses in TME (Ma et al., 2020). OV immunotherapy began in the 1950s when the virus was thought to invade and destroy cancer cells (Gebremeskel et al., 2021; Ang et al., 2022; Ripp et al., 2022). More recently, OVs have been at the forefront of cancer immunotherapy. Human adenovirus, the first and most potent virus, has been suggested for oncolytic immunotherapy (Mondal et al., 2020). Many studies have developed a number of strategies to stimulate antitumor immunity and to productively modulate the TME for potent and sustained anti-tumor immune cell activity (Workenhe and Mossman, 2014; Takasu et al., 2016; Guo et al., 2017; Mardi et al., 2022; Zhu et al., 2022). OVs may be armed to express T helper type 1 (Th1)-stimulatory cytokines/chemokines or co-stimulators to recruit and maintain potent antitumor immunity into the TME to focus their therapeutic activities at the site of the disease. The combination of OV with immunomodulatory drugs or antibodies that repair the TME has proven very promising in early studies. Furthermore, the combination of OV with other immunotherapeutic virotherapy (e.g. CAR T cells, equipped with bispecific T cell engagers, and prime-boost cancer vaccines) has also yielded promising initial findings (Ma et al., 2020).

## Oncolytic virus and immunotherapy

OVs can be as an adjunct to immune checkpoint inhibition (Ripp et al., 2022). OV has been combined with immune checkpoint inhibitors, with strong anti-tumor efficacy observed in pilot evaluations (Wang et al., 2020a; Christie et al., 2021; Ripp et al., 2022). Selective destruction of tumor tissues by OVs leads to antigen-agnostic enhancement of neoantigen-specific cytotoxic T cell responses, making OVs ideal companions for checkpoint blockade therapy (Russell and Barber, 2018). Systemic administration of tumor necrosis factor-armed myxoma virus plus immune checkpoint inhibitors eliminates lung metastatic murine osteosarcoma (Christie et al., 2021). Engineered OVs expressing a PD-L1 inhibitor activate tumor neoantigen-specific T cell responses (Wang et al., 2020a). Oncolytic activity of Zika virus requires cytotoxic T lymphocytes and is enhanced by immune checkpoint blockade against glioblastoma (GBM) (Nair et al., 2021). Chimeric antigen receptor (CAR) T-cells and OVs have emerged as promising cancer immunotherapies for cancer treatment (Watanabe et al., 2021). While immune checkpoint blockade has been approved for a wide range of solid tumors, other immunotherapies such as CAR T-cell and T-cell redirecting bispecific T-cell engagers (BiTEs) have been approved specifically for hematologic malignancies but less studied in solid tumors. BiTEs are an innovative class of immunotherapies that redirect T cells to tumor surface antigens and OV-BiTEs can serve as a blueprint for diverse OV-based cancer virotherapies (Heidbuechel and Engeland, 2021).

Other immunotherapies, such as cancer vaccines, cytokines, CAR T-cell therapy, small molecules, and OVs, are currently in use against colorectal cancer (Feng et al., 2021). Development of successful CAR T-cell therapy needs identification of specific tumor-associated antigens, as the primary target for CAR T-cell binding and activation. Many solid tumors carry well-characterized oncogenes which play a critical role in tumor cell proliferation, migration, and survival. These oncogenes are ideal targets for CAR T-cell therapy, particularly when the oncogene is expressed at low levels in normal tissues. More recently, it was found that interleukin (IL)-7-loaded oncolytic adenovirus elevates CAR T-cell therapy for GBM (Huang et al., 2021). The type 1 of HSV which expresses IL-12 efficiently replicates and kills human colorectal cancer cells (Haghighi-Najafabadi et al., 2021). IL-12 and granulocyte-macrophage colony-stimulating factor (GM-CSF) from an engineered oncolytic HSV have a synergistic effect, which boosts the immune response to enhance their antitumor effects in a B16-F10 murine melanoma model (Kim et al., 2021). Combination of IL-10 and oncolytic adenovirus demonstrated to enhance antitumor efficacy by cytotoxic T cells (Chen et al., 2021a). Avian virus, NDV, can induce tumor necrosis factor-alpha and augment its cytotoxicity as an antineoplastic agent (Lorence et al., 1988). Recently, IL-21 arming was reported to enhance the antitumor activity of OVV in monotherapy and combination immunotherapy (Chen et al., 2021b). Thus, OVV gene modification and cytokines expression can influence tumor killing, tumor infection, and immune response (Inoue et al., 2021).

Although OVs-derived type I interferons limit CAR T-cell therapy (Evgin et al., 2020), however, OVs-mediated expansion of bispecific CAR T cells can improve efficacy in solid tumors in mice (Evgin et al., 2022). OV-accompanied CAR T-cells are forced to overcome solid tumor challenges (Guedan and Alemany, 2018). Furthermore, the study by Xiao et al. have suggested that the CDK4/6 inhibition enhances OVs (VSVΔ51 and Zika virus) efficacy by T-cells activation and potentiating tumor-selective cells killing in refractory GBM (Xiao et al., 2022). Oncolytic H-1 parvovirus activates helper but not regulatory human CD4^+^ T cell responses (Moralès et al., 2012). Smac-Mimetics (SMs) have entered clinical trials in hematology and solid cancers, but unfortunately, results have been variable and limited (Morrish et al., 2020). OVs and SMs can synergistically drive anticancer T cell responses through complementary mechanisms (Kim et al., 2017). Oncolytic measles virus therapy enhances tumor associated antigens-CD8^+^ lymphocytes responses in patients with multiple myeloma (Packiriswamy et al., 2020). Interestingly, natural killer T cell immunotherapy combined with oncolytic VSV or MRV therapy significantly improved survival in mouse models of breast and ovarian cancer metastasis (Gebremeskel et al., 2021). HSV type 2-based OV acts as an attractant to guide the migration of adoptively transferred T cells to tumor sites (Fu et al., 2015). Recently, ORFV infection of lung cancer cells induced tumor cell oncolysis to release risk-associated molecular patterns and promoted dendritic cell maturation and CD8^+^ T cell infiltration in tumors by enhancing C-X-C motif chemokine ligand 16 (CXCL16) secretion (Wang et al., 2022). The report contributes to the understanding of the molecular mechanism of ORFV oncolysis and contributes to the development of new therapies for lung cancer (Wang et al., 2022). The immunosuppressive state of the breast cancer TME makes it difficult to treat with immunotherapy, but the chimeric oncolytic adenovirus armed with the chemokine regulated after activation, expressed and secreted by normal T cells (RANTES)/CC chemokine ligand 5 (CCL5) appears to be a promising drug candidate for the treatment of breast cancer (Ang et al., 2022).

Combination of oncolytic measles vaccine virotherapy and NK cell therapy results in enhanced oncolysis of A673 and HT1080 pediatric sarcoma cells (Klose et al., 2019). Recently, it was demonstrated that the combination of oncolytic herpes simplex 1-based-expressing human IL15/IL15Rα sushi domain fusion protein (named OV-IL15C) and off-the-shelf epidermal growth factor receptor (EGFR)-CAR NK cells triggers strong antitumor responses in GBM (Ma et al., 2021). This team demonstrated that OV-IL15C plus EGFR-CAR NK cells synergistically inhibit tumor growth and improve survival in comparison to monotherapy, correlating with increased intracranial infiltration and activation of NK and CD8^+^T cells and increased persistence of CAR NK cells in an immunocompetent model (Ma et al., 2021).

Mesenchymal stem cells along with OVs are joining forces against cancer (Moreno, 2021). Furthermore, combination of menstrual blood mesenchymal stem cells loaded with oncolytic adenovirus and peripheral blood mononuclear cells (PBMCs) enhanced antitumor efficacy (Moreno et al., 2019). Delivery of OVV found to match allogeneic stem cells and overcome key innate and adaptive immune barriers (Draganov et al., 2019). OV has been engineered to enforce leptin expression to reprogram tumor-infiltrating T-cell metabolism, thereby promoting tumor clearance (Rivadeneira et al., 2019). Immunomodulation by telomerase-specific oncolytic adenovirus synergistically enhances the antitumor efficacy of anti-programmed cell death 1 (PD-1) antibodies (Kanaya et al., 2020). Viro-antibody immunotherapy is also engineering OVs for genetic delivery of diverse antibody-based immunotherapies (Kontermann et al., 2021). Oncolytic HSV type 1 virus expressing full-length antibodies enhances the antitumor innate immune response against GBM and is capable of lysing tumor cells (Xu et al., 2021). Bispecific antibody (BsAb)-armed OVs can efficiently modulate TEM. BsAbs expressed by oncolytic HSV type 2 have been proved to convert heterologous T cells into homogeneous tumor-killing cells (Jin et al., 2022). Reshaping the TEM with OVs can blockade the immunosuppressive nonmetabolic circuitry, and positively regulates the immune synapse (Nguyen et al., 2022).

## Oncolytic viruses-induced immunogenic cell death

Apoptosis, pyroptosis, necrosis, ferroptosis and autophagy-dependent cell death belongs to cell death pathways identified so far, all of which have been classified as an ICD (Inoue and Tani, 2014). The ICD is a type of cancer cell death induced by certain physical and chemical therapies, such as OVs, chemotherapeutic drugs, radiation therapy and photodynamic therapy (Ahmed and Tait, 2020). OVs are naturally occurring or genetically engineered viruses that are administered intra-lesionally or intravenously to induce tumor cell death and activate antitumor immune responses. After entering tumor cells, OVs can induce innate and adaptive immune responses, thereby acting to eradicate cancer cells within the TME. Lysis of tumor cells can release pathogen-associated molecular patterns (PAMPs) such as viral nucleic acids and proteins, and damage-associated molecular patterns (DAMPs), such as heat shock proteins (HSPs) and high mobility group box 1b (HMGB1) stimulating innate immune response. NK cells and macrophages in the TME can recognize PAMPs/DAMPs through cell surface pattern recognition receptors (PRRs) to secrete inflammatory cytokines such as IFN-γ, IL-12, IFN-α, TNF-α, and IL-6, which can then induce antiviral and antitumor immune responses and recruit other innate immune cells from peripheral lymphoid organs (Saha et al., 2016). Furthermore, release of tumor-associated antigens or tumor-specific antigens and antigen presentation by antigen-presenting cells (APCs) following tumor cell lysis leads to adaptive immune responses and antigen-specific cytotoxic T cell and T helper cells. These tumor-reactive T cells can then induce ICD in tumor cells, a mechanism that has been demonstrated in preclinical study (Angelova et al., 2014).

OVs often induce ICD in the cancer cells, and they may interact directly with immune cells to trigger antitumor immunity (Workenhe and Mossman, 2014; Takasu et al., 2016; Mardi et al., 2022; Zhu et al., 2022). OVs engage with the immune system while they replicate within solid tumors. OVs replicate in cancer cells and release tumor antigens, which are perceived as dangerous because of simultaneous expression of PAMPs that activate APCs. Therefore, OVs provide the target antigens and danger signals required to induce adaptive immune responses. Oncolytic virotherapy and cancer ICD sharpen swords for improving cancer treatment strategies (Workenhe and Mossman, 2014). Alongside traditional ICD inducers such as anthracycline chemotherapeutics and radiation, OVs have emerged as new members of this class of therapeutics, which are increasingly promising outcomes in clinical trials involving cancer patient (Workenhe and Mossman, 2014). OVs are also attractive candidates for creating ICD, biological barriers limiting their success in the clinic, and groundbreaking strategies to potentiate ICD and antitumor immunity with rationally designed OV-based combination therapies (van Vloten et al., 2018). OVs have been engineered or combined with other ICD inducers to promote more efficient T cell cross-priming and, in many cases, to disrupt functional immune tolerance. Current therapeutic concepts against aggressive malignancies require induction of ICD characterized by exposure of calreticulin and release of ATP and HMGB1 in dying cells. A report by Koks et al. revealed that oncolytic NDV virotherapy induces long-term survival and tumor-specific immune memory in orthotopic glioma through the induction of ICD (Koks et al., 2015). Furthermore, a recent study has shown that oncolytic NDV elicits HMGB1, calreticulin exposure and, ATP secretion and HSP70/90 release, leading to ICD induction in melanoma cells (Shao et al., 2019). Recently, Wang et al. have revealed that an oncolytic NDV strain FMW (NDV/FMW) triggers the production and exposure of various ICD markers in prostate cancer cells, including HSP70/90, HMGB1, and calreticulin (Wang et al., 2020b). They also proposed that combining STAT3 inhibition with oncolytic NDV could enhance NDV-based anticancer effects in prostate cancer. Pancreatic ductal adenocarcinoma (PDAC) cells infected with oncolytic parvovirus H-1PV released only HMGB1, regardless of the nature of resulting cells whether those were non-dead cells, necrotic cells or cells that died by one of the programmed death pathways including moderate apoptosis (Angelova et al., 2014). It was found that complementary induction of ICD by oncolytic parvovirus H-1PV and gemcitabine in pancreatic cancer sustained induction of HMGB1 secretion raises the possibility that this response may be characteristic of a general alarm phenomenon (release of IL-1β suggests that a danger-sensing inflammasome platform may be involved) of H-1PV interaction with host cells (Angelova et al., 2014). Immune checkpoint inhibitors, such as anti-PD-1 antibodies, are able to improve clinical outcomes in certain cancers. Due to poor immune responses, PDAC is refractory to PD-1 blockade therapy. More recently, it has been demonstrated the p53-expressing telomerase-specific oncolytic adenovirus OBP-702 induces ICD and anti-tumor immune responses in human PDAC cells with different p53 expression statuses (Araki et al., 2022). Oncolytic OBP-702 adenovirus promotes ICD with secretion of extracellular adenosine triphosphate and HMGB1 by regulating p53-induced apoptosis and autophagy. Additionally, the OBP-702 significantly elevates the tumor infiltration of cytotoxic T cells and the anti-tumor efficacy of PD-1 blockade in a subcutaneous murine PDAC cells (PAN02) syngeneic tumor model (Araki et al., 2022).

OVs have an intrinsic ability to provide correct signals to trigger anti-tumor immune responses, both by delivering virus-derived innate signals and by ICD. It has also been that oncolytic HSV-1 induces ICD resulting in maturation of CD1c (BDCA-1)^+^ myeloid dendritic cells (Kalus et al., 2022). Molecules necessary for the induction of ICD are called DAMPs, which induce potent anti-cancer immunity. The oncolytic HSV-1 also can induce ICD in squamous cell carcinoma cells (Takasu et al., 2016). Oncolytic NDV-induced ICD release DAMPs, and causes resistance in malignancies. Oncolytic NDV has been demonstrated to induce autophagy-dependent immunogenic cell death in lung cancer cells (Ye et al., 2018). Furthermore, it has also been found that the STAT3 signaling contributes to oncolytic NDV-induced ICD in melanoma cells (Shao et al., 2019) and that targeting STAT3 enhances NDV-induced ICD in prostate cancer cells (Wang et al., 2020b). Talimogene laherparepvec (T-VEC) is a modified HSV to selectively grow in tumor cells and express the immunostimulatory transgenic GM-CSF (Hu et al., 2006). The mechanism of action of T-VEC has not been fully elucidated, however it is plausible that the induction of ICD and activation of host anti-tumor immunity may activate T-VEC. A recent report suggested that OVs immunotherapy induces ICD and overcomes stimulator of interferon genes deficiency in melanoma (Bommareddy et al., 2019).

Current treatments fail to significantly improve patient survival, which is often limited to less than one-year post diagnosis. Virotherapy, based on the use of OVs, exerts an anticancer effect through direct cell lysis and OVs dl 922-947 triggers ICD in mesothelioma, reducing xenograft growth (Di Somma et al., 2019). The CAR T-cell therapies are a promising and rapidly expanding treatment option for a variety of human malignancies. Despite continued progress in CAR T-cell therapies in hematological malignancies, application of this therapeutic strategy in solid tumors has been hampered by antigenic heterogeneity, suboptimal CAR T-cell trafficking, and the immunosuppressive features of the TME. Oncolytic virotherapy is a new type of cancer therapy that uses competent or genetically modified OVs to preferentially proliferate in tumor cells. Combining OV with CAR T cells is a promising candidate to overcome the current shortcomings of CAR T-cell application in tumors by triggering ICD in cancer cells (Mardi et al., 2022). OVs therapy leads to ICD of virus-infected tumor cells and this has been shown in preclinical models to enhance the CD8^+^ T cell response against tumor-associated antigens, leading to enhance tumor antigen-specific T-cell responses in patients with multiple myeloma (Packiriswamy et al., 2020). In addition to direct oncolysis, OVs trigger ICD and primes antitumor immunity. Ye et al. have showed that oncolytic NDV induces autophagy-dependent ICD in lung cancer cells (Ye et al., 2018). OVV synergizes with doxorubicin in inducing ICD in platinum-resistant ovarian cancer cells and increases survival in syngeneic and xenograft tumor models (Mistarz et al., 2021). In another study, Ma et al. have demonstrated that wild-type adenovirus, semliki forest virus and OVV can induce various ICD and also stimulate antitumor immune responses (Ma et al., 2020).

## Oncolytic virus-regulated induction of apoptosis of tumor cells

OVs are a novel therapeutic approach that works by activating immune function and inducing tumor cell apoptosis (Li et al., 2022a). As classical OVs studies rely heavily on their natural oncolysis, the discovery of tumor-selective virus-mediated apoptosis marks the appeal of an alternative cancer therapy in the form of OVs (Kalyanasundram et al., 2018). The replication ability of OVs in normal cells is low, while the virus can multiply specifically in tumor cells, which are lysed by the proliferation of the virus. OVs are described as naturally occurring or genetically engineered viruses that specifically replicate and induce apoptosis in cancer cells but not in normal cells (Fukuhara et al., 2016).

OV initiates targeted infection and induces tumor cell apoptosis while expressing therapeutic transgenes such as checkpoint inhibitors, cytokines, tumor antigens in the tumor (Rommelfanger et al., 2013). OVs immunotherapy and proteasome inhibition are two emerging targeted cancer therapies. It has been demonstrated that Bortezomib, a proteasome inhibitor, disrupts protein degradation in cells, leading to accumulation of unfolded proteins and induction of apoptosis (Aspirin et al., 2021). Wu et al. constructed an OV-like nanoplatform that degrades in an acidic tumor environment to release Poly IC and Zn^2+^. Importantly, released Poly IC has OV-like function and induces tumor cell apoptosis (Wu et al., 2022).

OVs can induce intracellular redistribution of Ras to promote apoptosis and progeny virus release (Garant et al., 2016). HSV, adenovirus and alphavirus mediate the induction of apoptosis for anti-prostate cancer (Lu et al., 2021). Oncolytic specificity of NDV is mediated by selectivity against apoptotic cells (Mansour et al., 2011). Discovery of tumor-selective virus-mediated apoptosis marks the emerge of an alternative cancer therapy using NDV strain AF2240 as an OV format (Kalyanasundram et al., 2018). Recently, an oncolytic measles virus encoding IL-12 treats colon cancer *in vivo* and *in vitro* to study its effect on colon cancer cell viability and apoptosis (Haghighi-Najafabadi et al., 2021). Turpin et al. illustrate the duality of ZIKA Virus-controlled apoptotic effects depending on whether it occurs (Turpin et al., 2022). Lal and Rajala developed a recombinant measles virus carrying BNiP3, a pro-apoptotic gene of human origin, as an oncolytic agent and demonstrated its ability to induce apoptosis in breast cancer cells *in vitro* (Lal and Rajala, 2018). Oncolytic goat herpesvirus 1 induces apoptosis in mesothelioma cell lines and synergizes with cisplatin as a new potential immunotherapy (Forte et al., 2021). IL-24 is an important cytokine that belongs to the family of activating caspases and promotes the repression of STAT3 when cells enter the apoptotic pathway. OVV carrying the IL-24 gene inhibits the growth of lung cancer by inducing apoptosis (Lv et al., 2016). Hypoxia- and telomerase-responsive oncolytic adenoviruses expressing secreted trimeric tumor necrosis factor-related apoptosis-inducing ligand (TRAIL) trigger tumor-specific apoptosis and promote virus spread in TRAIL-resistant GBM (Oh et al., 2018). Furthermore, TRAIL-armed oncolytic poxvirus inhibits lung cancer cells by inducing apoptosis (Hu et al., 2018). NDV decreased mitochondrial membrane potential, suggesting an intrinsic pathway of apoptosis in oral cancer cells (Morla et al., 2019).

An early study showed that oncolytic VSV induces apoptosis by modulating the PKR, Fas, and Daxx pathways (Gaddy and Lyles, 2007). Matrix protein mutants of VSV are promising oncolytic agents for cancer therapy. Lal and Rajala have developed a recombinant measles virus harboring BNiP3, a pro-apoptotic gene of human origin, which as an oncolytic agent, has been shown to induce apoptosis in breast cancer cells *in vitro* (Lal and Rajala, 2018). Recently, M51R and Delta-M51 matrix proteins of VSV induce apoptosis in colorectal cancer cells (Gray et al., 2019). Proto-parvovirus H-1 induces lytic infection and apoptosis *in vitro* but fails to improve survival *in vivo* (Lacroix et al., 2018).

## Oncolytic viruses-modulated cellular autophagy machinery in infected tumor cells

Upon infection, OV disrupts the autophagy machinery in infected tumor cells both *in vitro* and *in vivo.* The recent literature on targeting autophagy with either the autophagy inducers, such as rapamycin (Meng et al., 2013). Autophagy can crosstalk with OVs in cancer therapy (Jin et al., 2021) and induction of autophagy by OVs plays a dual role in GBM (Kamynina et al., 2021). Beclin-1 has been well characterized to play an important role in autophagy, the main catabolic pathway by which cells degrade macromolecules and damaged organelles. Importantly, Beclin1-armed OVV enhances the efficacy of first-line therapy with rituximab, cyclophosphamide, eirenicon, vindoline, and prednisolone against lymphoma *in vitro* and *in vivo* (Xie et al., 2021). Enhancement of therapeutic efficacy of OVV-armed with Beclin-1 was also seen in myeloma and leukemia (Lei et al., 2020). Oncolytic MRV has been demonstrated to induce autophagy in KRAS-mutated colorectal cancer (Jiffry et al., 2021).

Interestingly, oncolytic paramyxoviruses have been found to induce autophagy and lead to tumor cell death rather than their survival. Oncolytic paramyxovirus induces autophagy as a discreet weapon in cancer therapy (Keshavarz et al., 2019). Deficiency in the inositol-requiring transmembrane kinase/endonuclease-1(IRE1α)-autophagy axis enhances the antitumor effect of oncolytic virus M1, a naturally occurring alphavirus. (Li et al., 2018). Furthermore, the class III phosphatidylinositol 3-kinase/Beclin-1 pathway plays a role in NDV-induced autophagy and virus production. Oncolytic NDV induces autophagy in U251 glioma cells to elevate virus replication (Meng et al., 2012). It was approved that pharmacological modulation of autophagy identified to enhance NDV-mediated oncolysis in drug-resistant lung cancer cells (Jiang et al., 2014). Autophagy participates in gastric adenocarcinoma cell death induced by recombinant NDV *in vitro* (Bu et al., 2015). Oncolytic NDV induces cell death of lung cancer spheroids and is further enhanced by pharmacological suppression of autophagy (Hu et al., 2015). Recently, the matrix protein of VSV has been shown that can cause autophagy-induced cell death in the breast cancer (Askari et al., 2021).

Mre11, the core of the Rad50/Nbs1/Mre11 complex, is one of key DNA damage response proteins. Inhibition of Mre11 by oncolytic adenovirus is associated with autophagy and synergizes with ionizing radiation (Rajecki et al., 2009). Rodriguez-Rocha et al. demonstrate that adenovirus-induced autophagy is positively correlated with viral replication and oncolytic cell death, and that autophagy may generate nutrients that can be used to construct viral progeny particles (Rodriguez-Rocha et al., 2011). Suppression of autophagy increases the effects of E1A-defective oncolytic adenovirus dl922-947 against glioma cells both *in vitro* and *in vivo* (Botta et al., 2012). Oncolytic adenovirus-induced autophagy exhibits a tumor-suppressive immunotherapy effect (Tazawa et al., 2013). Oncolytic adenoviruses and certain chemotherapeutic drugs induce autophagy and immunogenic cancer cell death. Temozolomide-containing oncolytic adenovirus induces autophagy and antitumor immune responses in cancer patients (Liikanen et al., 2013). A study by Cheng et al. evaluated a unique combination of novel adenovirus-cycE (a novel E1b-deleted oncolytic adenovirus-cycE, in which Ad E1a gene is driven by the cyclin E promoter) with rapamycin, an autophagy inducer and first-line chemotherapy drug, suggesting that combination of autophagy inducer rapamycin and oncolytic adenovirus enhances antitumor effect of cancer cells (Cheng et al., 2013). Oncolytic adenoviruses, such as Delta-24-RGD, are replication-competent viruses that have been genetically engineered to induce selective cancer cell lysis. Oncolytic adenovirus-mediated autophagy has been shown to require the C-Jun N-terminal kinase (Klein et al., 2015). Oncolytic adenovirus Delta-24-RGD induces extensive glioma prototypical remodeling during autophagy (González-Morales et al., 2019). Furthermore, recombinant adenoviruses expressing apoptin inhibits the growth of MCF−7 breast cancer cells and influences cell autophagy (Chen et al., 2019).

## Oncolytic viruses-modulated apoptosis, autophagy, syncytium formation in cancer cell lines

In addition to direct oncolysis, OV induces ICD and elicits antitumor immunity. Recently, it has been reported that recombinant Chinese measles virus vaccine strain rMV-Hu191 inhibits the growth of human colorectal cancer by inducing autophagy and apoptosis *via* PI3K/Akt pathway (Zhang et al., 2021). RGD (Arg-Gly-Asp)-modified oncolytic adenovirus carrying shPKM2 (M2 isoform of pyruvate kinase) (OAd.R.shPKM2) exhibits potent cytotoxicity in pancreatic cancer by inhibiting autophagy and promoting apoptosis (Xu et al., 2017). A novel dual regulatory oncolytic adenovirus (Ad.wnt-E1A(△24bp)-TSLC1) targets Wnt signaling to efficiently inhibit cancer-like cell growth through apoptosis, autophagy and metastasis in a HCC model (Zhang et al., 2017). Oncolytic adenovirus AdΔΔ attenuates sensitivity to mitoxantrone-induced apoptosis *via* Bcl-2-dependent autophagy (Aguirre-Hernández et al., 2018). Furthermore, Araki et al. have demonstrated that oncolytic adenovirus OBP-702 promotes ICD with secretion of extracellular adenosine triphosphate and high-mobility group box protein B1 by regulating p53-induced apoptosis and autophagy. The OBP-702 significantly elevates the tumor infiltration of CD8^+^ T cells and the anti-tumor efficacy of PD-1 blockade in a subcutaneous PAN02 syngeneic tumor model (Araki et al., 2022). Japanese enveloped hemagglutination virus induces apoptosis and autophagy in human prostate cancer PC3 cells (Qian et al., 2018). Inactivated Sendai virus (ISV) was found to induce ROS-dependent apoptosis and autophagy in human prostate cancer cells (Qian et al., 2018). ISV strain Tianjin also induces apoptosis and autophagy through generation of reactive oxygen species in osteosarcoma MG-63 cells (Han et al., 2019).

The oncolytic NDV is an avian paramyxovirus, which can replicate in many tumor types and exert strong cytotoxic effects (Zamarin and Palese, 2012; Buijs et al., 2014; Ganar et al., 2014; Schirrmacher and Fournier, 2014; Zamarin et al., 2014; Cuadrado-Castano et al., 2015; Schirrmacher, 2015). NDV can induce ICD, apoptosis, autophagy-related cell death, necroptosis in a range of cancers (Hu et al., 2015; Ye et al., 2018; Shao et al., 2019; Mozaffari Nejad et al., 2020; Wang et al., 2020b). Oncolytic NDV has displayed potent anti-tumor activities both in preclinical studies and in clinical trials (Ganar et al., 2014; Zamarin et al., 2014; Cuadrado-Castano et al., 2015; Schirrmacher, 2015; Tayeb et al., 2015; Schirrmacher, 2016). Oncolytic NDV may be effective in the treatment of lung cancers, as its natural tropism is the respiratory tract of avian species. Hu et al. have shown that the oncolytic NDV triggers cell death of lung cancer spheroids and is enhanced by pharmacological inhibition of autophagy (Hu et al., 2015). Oncolytic NDV is a potent ICD-inducer and that autophagy contributes to NDV-mediated induction of ICD in lung cancer cells (Ye et al., 2018). Oncolytic effect of NDV Hitcher B1 strain on cervical cancer cell proliferation is mediated by increased expression of cytochrome c, apoptosis and autophagy pathways (Mozaffari Nejad et al., 2020). Another avian virus, ARV, is not associated with human diseases, and preexisting immunity would not hinder its clinical application (Cai et al., 2019). It has been used in anti-cancer research (Benavente and Martínez-Costas, 2007; Chiu et al., 2018; Cai et al., 2019; Manocha et al., 2021a; Manocha et al., 2021b; Li et al., 2022b). ARVs have several unique features that are different from MRV. They can induce syncytia to facilitate virus spread and distribution within a tumor. ARV was originally thought to act mainly through apoptosis (Shih et al., 2004; Chulu et al., 2007; Lin et al., 2011; Brown et al., 2018). The RhoA/ROCK1 pathway is known to regulate oncolytic ARV-mediated switch from autophagy to apoptosis (Lin et al., 2015). Several reports have suggested that ARV-induced apoptosis is through p53 and mitochondria-mediated pathway, and p53 is regulated by mitogen-activated protein kinases and protein kinase Cδ during ARV S1133-induced apoptosis (Chulu et al., 2007; Lin et al., 2009). Interestingly, the PI3-kinase/Akt/NF-κB and STAT3 signaling can be activated in the early stages of ARV infection which results in an inflammatory response and delayed apoptosis (Lin et al., 2010). Oncolytic ARV may combine with MRV due to their ability to evade pre-existing immunity. Oncolytic ARV σC protein is an apoptosis inducer which induces apoptosis (Shih et al., 2004), whereas p10 causes cytopathic effect (CPE) in mammalian and cancer cell lines (Liu et al., 2008). An earlier study suggested that p10 mediates syncytium formation through the RhoA and Rac1-dependent signaling pathway (Liu et al., 2008). It was also reported that membrane-proximal basic residues, transmembrane glycine residues, and palmitoylation in the p10 protein are necessary for the formation of syncytium (Shmulevitz et al., 2003). More recently, it has been demonstrated that chitosan-based delivery of ARV p10 gene (ARV-p10 CH-NPs) is able to induce cell fusion in cultured melanoma cells, exhibiting a mild cytotoxic effect. Importantly, intratumor injection of ARV-p10 CH-NPs delayed tumor growth, without altering lymphoid populations in the spleen and tumor tissue (Robles-Planells et al., 2020). Recently, Jeon and Jung reported that the use of the murine leukemia virus (MLV)-based replication-competent retroviral (RCR) vector engineered to express the p10 fusion protein of Pulau virus in human cancer cell lines, inducing syncytium formation (Jeon and Jung, 2022). Thus, the RCR vector carrying p10 is a promising candidate for gene therapy for cancer. The nonstructural protein p17 of ARV is capable of modulating autophagy by regulating multiple signaling pathways, benefiting virus replication (Chi et al., 2013; Huang et al., 2015; Chiu et al., 2016; Huang et al., 2017; Chiu et al., 2018; Chiu et al., 2019; Huang et al., 2022b; Li et al., 2022b). ARV p17 also modulates cell cycle, viral protein synthesis, virus replication, and host cellular translation (Chi et al., 2013; Huang et al., 2015; Chiu et al., 2016; Huang et al., 2017; Chiu et al., 2018; Chiu et al., 2019; Huang et al., 2022b; Li et al., 2022b). Since p17 induces autophagy by triggering PKR/eIF2α signaling pathways accompanied by suppression of Akt and mTORC1(Chi et al., 2013), it triggers the innate immune system and can mount a potent immune response against tumors (Cai et al., 2019; Chiu et al., 2019). Recent reports have revealed that p17 retards cell cycle of several cancer cell lines and reduces tumor size *in vivo* (Chiu et al., 2018), and suppresses angiogenesis by promoting DPP4 secretion (Manocha et al., 2021a). More recently, it was demonstrated that ARV p17 is a HSP90 client protein that can regulate the formation of the HSP90/Cdc37 chaperone complex to increase its stabilization and enhance the synthesis and accumulation of viral structural proteins σA and σC in viral factories for virus assembly (Huang et al., 2022a; Huang et al., 2022b). The σA protein of ARV enhances glycolysis and the TCA cycle and regulate cellular fatty acid metabolism to produce more ATP for virus replication in mammalian and human cancer cell lines (Chi et al., 2018; Hsu et al., 2022; Hsu et al., 2023).

Collectively, OVs have advanced as promising anti-cancer immunotherapies by exploiting the apoptosis and autophagy pathways which underline the success of immunotherapy approaches.

## Clinical trials

OV is using as an anticancer agent in preclinical and clinical trials. Clinical trials have been conducted in the 1950s using wild-type and non-engineered *in vitro*-passaged virus strains and OV vaccine strains (Watanabe and Goshima, 2018). Using OVs to treat cancer is promising with clinical trial results (Malogolovkin et al., 2021). A variety of OVs including genetically engineered and natural viruses have shown promise in preclinical models and clinical studies. In 2005, China Food and Drug Administration (FDA) approved its first OV drug Oncorine (H101) and H101 based on human adenovirus 5 has been approved for human clinical trials (Shi and Zheng, 2009). OVV has also been systematically explored as an OV past 20 years. Of the three OVVs tested cancer patients, Pexa-Vec (JX-594) demonstrated clinical development of this OV, currently undergoing evaluation in a global Phase III clinical trial in patients with hepatocellular carcinoma (Guo et al., 2019).

The safety and efficacy of a proprietary variant of Coxsackievirus A21 (Cavatak™) (Au et al., 2005) administered intratumorally or intravenously as a stand-alone immunotherapeutic agent has been demonstrated in 57 patients with the unresectable stage IIIC-IV melanoma, and 30 individuals with other advanced solid tumor malignancies (Pandha et al., 2015). ECHO-7 strain Rigvir^®^ belongs to the Enterovirus genus of Picornaviridae family. Rigvir^®^ is a non-pathogenic oncolytic virus selected and engineered for melanoma, which was originally isolated from the gastrointestinal tract of healthy children without genetic modification. Rigvir^®^ registered in Latvia in 2004 for the treatment of melanoma over a decade ago (Kelly and Russell, 2007). ONCOS-102 (a human serotype 5 adenovirus expressing GM-CSF optimized for replication in malignant cells, also known as Ad5/3-D24-GMCSF or CGTG-102 (Bramante et al., 2014; Ranki et al., 2014). The clinical profile was studied in nine melanoma subjects who received ONCOS-102 in combination with standard of care chemotherapy (Bramante et al., 2015). In two cohorts, 15 patients with sarcoma and 90 subjects with GM-CSF-sensitive tumors received ONCOS-102 as a stand-alone immunotherapy intervention (Bramante et al., 2014). The safety and efficacy of GL-ONC1, a genetically modified OVV also known as GLV-1h68, has been evaluated in 14 patients with malignant pleural effusions who received intrapleural GL-ONC1 as a stand-alone immunotherapy and intervention 19 subjects affected by head-neck cancer who received GL-ONC1 intravenous therapy in combination with cisplatin-based chemoradiation (Vacchelli et al., 2013).

A phase I clinical trial with a second-generation oncolytic HSV expressing GM-CSF (Onco VEXGM-CSF) was conducted to determine the safety of the virus, seek evidence of biological activity, and determine dosage regimen for future studies. It was found that the phase I study of OncoVEXGM-CSF, a second-generation oncolytic HSV expressing GM-CSF in 2006. Onco VEXGM-CSF was well tolerated and could be administered safely using the multiple dose regimen described. Evidence for antitumor effects was seen (Hu et al., 2006). This renewed interest in OVs led to the approval of the first OV, T-VEC based on HSV type 1, in 2015 by the US FDA and the European Medicines Agency (EMA) (Harrington et al., 2015). On October 27, 2015, the U.S. FDA officially approved T-VEC for the treatment of melanoma patients with injectable but unresectable lesions in the skin and lymph nodes. Imlygic^®^ (T-VEC) commercialized by Amgen, Inc. thus became the first OV approved for cancer treatment in the United States (Pol et al., 2016). Cancer vaccines and OVs virotherapy are promising therapeutic strategies that have the potential to provide greater clinical benefit to patients with advanced cancer (Guo et al., 2019). OV agent T-VEC has been approved by the US FDA for the treatment of unresectable melanoma with limited visceral metastasis (Ripp et al., 2022).

The use of cyclophosphamide as an immune modulator in phase I clinical trial can optimize oncolytic MRV being delivered to solid tumors (Roulstone et al., 2015). MRV displays tropism and replicates efficiently in tumor cells with the activated Ras pathway. This allows the application of MRV in immunotherapy, either alone or in combination with conventional and unconventional treatments, such as the synergistic cytotoxicity of MRV in combination with cisplatin-paclitaxel dual chemotherapy (Roulstone et al., 2013). MRV has been used in cancer immunotherapies under the name REOLYSIN^®^. This formulation of MRV has been tested in preclinical and phase I-III clinical trials across a broad range of cancer indications (Roulstone et al., 2013). Oncolytic HSV type 1 combination with lenalidomide has been applied to treat plasma cell neoplasms (Oku et al., 2021). OV is a new option emerging through systemic delivery of murine LIGHT (TNFSF14/CD258)-armed myxoma virus for the treatment of advanced syngeneic murine lung metastatic osteosarcoma (Christie et al., 2022). Oncolytic adenovirus Ad5/3-Δ24aCTLA4 stimulates T cells from cancer patients for clinical trials (Dias et al., 2012). Improving CAR T-cell therapy for solid tumors by using OV-driven production of a bispecific T cell engager (BiTE) could be further evaluated in clinical trials (Wing et al., 2018). Adenovirus Delta-24-RGD shows significant efficacy in phase I clinical trial of glioblastoma (GBM) (González-Morales et al., 2019). Mesothelin-redirected CAR T Cell therapy shows efficacy as antitumor agent in clinical trials (Watanabe et al., 2018). Clinical trials of combination therapies are underway, focusing on the association of immune checkpoint inhibitors with chemotherapy, with encouraging results, especially in the early stages with pembrolizumab and doxorubicin, anti-angiogenic agents and immune checkpoints inhibitors or synergy with OVs (Roulleaux Dugage et al., 2021). Teserpaturev/G47Δ (Delytact^®^) is a third-generation recombinant oncolytic HSV-1 developed by Daiichi Sankyo Corporation for the treatment of certain solid cancers. Teserpaturev/G47Δ has been approved in Japan for the treatment of malignant glioma and is currently in clinical trials for the treatment of prostate cancer (Phase II), malignant pleural mesothelioma (Phase I) and recurrent olfactory neuroblastoma (Phase I) development stage leading to the first approval for the treatment of malignant glioma (Frampton, 2022). Current status of clinical trials discussed in this review are outlined in **Table 1**.

**Table 1:** Clinical trials in this mini review.

OV agent	Stage of development	Mode of action	Reference
Talimogene laherparepvec (T-VEC)	Approved by the U.S. FDA	For the treatment of unresectable melanoma with limited visceral metastases	Ripp et al., 2022
Imlygic^®^	Approved by the U.S. FDA	Talimogene laherparepvec (T-VEC, also known as OncoVEXGM-CSF) for the treatment of melanoma patients with injectable but unresectable lesions in the skin and lymph nodes.	Pol et al., 2016
T-VEC	Approved by the US FDA and the European Medicines Agency (EMA)	HSV modified to selectively grow in tumor cells and express the immunostimulatory transgenic granulocyte-macrophage colony-stimulating factor	Harrington et al., 2015
Onco VEXGM-CSF	Phase I clinical trial	A second-generation oncolytic HSV expressing GM-CSF	Hu et al., 2006
Cavatak™	Treatment of unresectable stage IIIC-IV melanoma and other advanced solid tumor malignancies	The safety and efficacy of a proprietary variant of Coxsackievirus A21	Au et al., 2005; Pandha et al., 2015
Rigvir^®^	Registered in Latvia in 2004 for the treatment of melanoma	ECHO-7 strain Rigvir^®^ belongs to the Enterovirus of Picornaviridae family. Rigvir^®^ originally isolated from the gastrointestinal tract of healthy children without genetic modification.	Kelly and Russell, 2007
ONCOS-102	Treatment of melanoma in combination with standard of care chemotherapy, sarcoma and GM-CSF-sensitive tumors	A human serotype 5 adenovirus expressing GM-CSF optimized for replication in malignant cells, also known as Ad5/3-D24-GMCSF or CGTG-102	Bramante et al., 2014; Ranki et al., 2014; Bramante et al., 2015
GL-ONC1	Treatment of malignant pleural effusions and head-neck cancer in combination with cisplatin-based chemoradiation	A genetically modified OVV also known as GLV-1h68	Vacchelli et al., 2013
Oncolytic MRV	Phase I clinical trial	The use of cyclophosphamide as an immune modulator optimize MRV being delivered to solid tumors	Roulstone et al., 2015
REOLYSIN^®^	Preclinical stage and phase I-III clinical trials	This formulation of the human reovirus in a broad range of cancer indications	Roulstone et al., 2013
Oncolytic HSV type 1	Treatment of plasma cell neoplasms	HSV type I combination with lenalidomide	Oku et al., 2021
Myxoma virus	Therapeutic for later-stage syngeneic murine lung metastatic osteosarcoma	Options by systemic delivery of murine LIGHT (TNFSF14/CD258)-armed myxoma virus	Christie et al., 2022
Oncolytic adenovirus Ad5/3-Δ24aCTLA4	Testing the approach in clinical trials	Stimulation of T cells of cancer patients by oncolytic adenovirus Ad5/3-Δ24aCTLA4	Dias et al., 2012
CAR T cells therapy of solid tumors with oncolytic virus	Further evaluated in clinical trials	Driven production of a bispecific T-cell engager (BiTE)	Wing et al., 2018
Adenovirus Delta-24-RGD	Phase I clinical trial	Adenovirus Delta-24-RGD has shown a remarkable efficacy for glioblastoma	González-Morales et al., 2019
Oncorine (H101)	Approved by China FDA	The first OV drug, the second gene therapy-based medicine, for treatment of advanced head and neck cancer	Shi and Zheng, 2009
Pexa-Vec (JX-594)	Phase III clinical trial	OVVs tested cancer patients, Pexa-Vec (JX-594) demonstrated clinical development of this OV in patients with hepatocellular carcinoma	Guo et al., 2019
Teserpaturev/G47Δ (Delytact^®^)	Clinical trials for the treatment of prostate cancer (Phase II), malignant pleural mesothelioma (Phase I), and recurrent olfactory neuroblastoma (Phase I)	Third-generation recombinant oncolytic herpes simplex virus type I developed by Daiichi Sankyo Corporation for the treatment of certain solid cancers	Frampton, 2022

## Conclusion

OVs destroy cancer cells by inducing multiple cell death pathways. These include apoptosis, necroptosis, autophagic cell death, and pyroptosis, each of which is a major form of death for specific OVs. OV-induced cancer cell death is mainly immunogenic and has the potential to elicit antitumor immune responses (Guo et al., 2014). The current progress of OVs in cancer treatment is the focus of this min-review. Their interaction with the immune system, ICD, apoptosis, and autophagy as well as new strategies in their virotherapy efficacy have been reviewed. OVs are antigen-agnostic cancer vaccines (Russell and Barber, 2018) and novel strategies for cancer treatment. OVs virotherapy are alternative promising therapeutic approaches against multiple chemo-resistant and radiation-resistant cancers (Fukuhara et al., 2016; Guo et al., 2017; Forte et al., 2021). Recombinant OVs are novel concepts to reduce the side effects of systemic cancer treatment while enhancing the oncolytic properties. An array of research efforts is ongoing in improving the virotherapy with OVs and these approaches hold great future promises to handle ever increasing issues of cancers.

## Author contributions

Y-YW and H-JL wrote the paper, H-JL supervised the project. H-JL,T-KS, M-SC, and MM revised and edited the manuscript. All authors contributed to the article and approved the submitted version.
